# Skeletal, Dental and Soft Tissue Cephalometric Changes after Orthodontic Treatment of Dental Class II Malocclusion with Maxillary First Molar or First Premolar Extractions

**DOI:** 10.3390/jcm11113170

**Published:** 2022-06-02

**Authors:** Johan Willem Booij, Marco Serafin, Rosamaria Fastuca, Anne Marie Kuijpers-Jagtman, Alberto Caprioglio

**Affiliations:** 1 Private Practice in Gorinchem, 4207 AC Gorinchem, The Netherlands; mrbooij@planet.nl; 2 Department of Biomedical Sciences for Health, University of Milan, 20133 Milan, Italy; 3 Private Practice in Varese, 21100 Varese, Italy; rosamaria.fastuca@gmail.com; 4 Department of Orthodontics and Dentofacial Orthopedics, School of Dental Medicine, Medical Faculty, University of Bern, CH-3010 Bern, Switzerland; a.m.kuijpers-jagtman@umcg.nl; 5 Department of Orthodontics, University Medical Center Groningen, University of Groningen, 9713 GZ Groningen, The Netherlands; 6 Faculty of Dentistry, Universitas Indonesia, Jakarta 10430, Indonesia; 7 Department of Biomedical, Surgical and Dental Sciences, Section of Orthodontics, University of Milan, 20122 Milan, Italy; alberto.caprioglio@unimi.it; 8 Fondazione IRCCS Ca Granda, Ospedale Maggiore Policlinico, 20122 Milan, Italy

**Keywords:** class II malocclusion, maxillary first premolar extraction, maxillary first molar extraction, orthodontic camouflage, orthodontic therapy

## Abstract

The aim of the present retrospective study was evaluating skeletal, dental and soft tissue changes of two groups of Class II patients orthodontically treated with extractions of upper first premolars (U4 group) and upper first molars (U6 group). In total, 21 patient records (9M and 12F; mean age 12.5 ± 1.2 years) were selected for the U4 group, and 38 patient records (17M and 21F; mean age 13.2 ± 1.3 years) were recruited for the U6 group. Twenty cephalometric variables were analysed on standardised lateral cephalograms at baseline (T0) and at the end of orthodontic treatment (T1). Means and standard deviations (SDs) were calculated for both groups and increments were calculated. After revealing the normal distribution of data with the Shapiro–Wilk test, Student’s *t*-test was used to compare variables at T0 between groups. A paired *t*-test was used to analyse changes between time points within each group, and Student’s *t*-test to compare differences between groups at T1. Both groups showed a significant increase in the distance among upper second molars and the vertical pterygoid line (PTV-maxillary second molar centroid U6 group: 6.66 ± 5.00 mm; U4 group: 3.66 ± 2.20 mm). Moreover, the distance of upper incisors to the palatal plane significantly increased (PP-maxillary incisor tip U6 group: 1.09 ± 1.52 mm; U4 group: 0.20 ± 2.00 mm; *p* = 0.061). Significant changes were found for overjet (U6 group: −4.86 ± 1.62 mm; U4 group: −3.27 ± 1.90 mm; *p* = 0.001). The distance between upper lip and esthetic plane showed a significantly reduction in both groups (ULip-E Plane U6 group: −2.98 ± 1.65 mm; U4 group: −1.93 ± 1.57 mm). No statistically significant changes were found in sagittal or vertical skeletal values. The significantly larger reduction of upper lip protrusion and overjet in the U6 group compared to the U4 group suggests preferring molar extraction treatment for severe Class II with protrusive soft tissues’ profile and increased overjet. Since no differences on vertical values were found, an increased SN^GoGn angle should not be considered a discriminating factor for choosing molar extraction treatment.

## 1. Introduction

Several treatment approaches have been proposed to manage an Angle Class II malocclusion, taking into consideration skeletal relationship, dentoalveolar discrepancies and residual facial growth [1]. Extraction strategies should consider the patient’s soft tissue, sagittal and vertical discrepancy, growth pattern and crowding, in order to improve or, at least, not worsen esthetic, occlusal and functional relationships [2,3,4].

Extraction of upper premolars is a valid choice to mask skeletal anomalies through dental compensation [5] and solve upper anterior malalignment or excessive teeth proclination in the absence of crowding in the mandibular arch [6]. Moreover, obtaining a Class II molar relationship is easier than obtaining a Class I, which requires the patient’s compliance during anchorage management [7], while lower incisor proclination due to treatment is minimal [8]. In addition, the treatment time is shorter because obtaining a Class I molar relationship is generally more challenging [9]. Finally, this treatment approach promotes dentoskeletal and tissue alterations with a consequent improvement of the relationship between the bony bases and soft tissue profile [10].

Another solution to mask sagittal discrepancy is maxillary first molar extraction, which is recommended in the case of severe carious decay, enamel hypoplasia, difficult endodontic or periodontal problems [11,12]. This treatment might be preferred in patients with expected poor compliance, healthy second and third maxillary molars and a long-face facial type [13]. This approach ensures a good result for the sagittal dimension and good post-treatment esthetics [14]. Upper incisor retraction mainly improves the overjet, and a slight mandibular growth may take place due to a bite-closure effect [15,16].

Both extraction treatment strategies improve Class II malocclusion with general good results in dentoskeletal changes and esthetic management. Nevertheless, no direct comparative studies were performed between these two treatment strategies in order to understand different results and indications of them. The aim of the present cephalometric investigation was, therefore, to compare skeletal, dental and soft tissue changes in two groups of Class II patients treated with extractions of upper first premolars (U4 group) and extractions of upper first molars (U6 group). The null hypothesis was that there were no differences between groups, regardless of the performed treatment.

## 2. Materials and Methods

### 2.1. Sample Selection

The initial sample of the present retrospective study consisted of 48 Class II Division I patient records treated with upper first premolar extractions and 97 Class II Division I patient records treated with upper first molar extractions; the same trained operator selected both groups from two private practices between the years 2018–2021. The study protocol was reviewed and approved by the Ethical Committee of the University of Milan (approval number ROS18/02) and the University Medical Center Groningen (approval number METc 2020/460), and all the procedures adhered to the World Medical Organization Declaration of Helsinki; an informed consent was obtained from all subjects involved in the study.

From the initial sample of 48 patients, 21 patients (9M, 12F; mean age 12.5 ± 1.2 years) treated by the same orthodontist (A.C.), were selected for the premolar extraction treatment group (U4 group). From the initial sample of 97 patients, 38 patients (17M, 21F; mean age 13.2 ± 1.3 years) treated by the same orthodontist (J.W.B), were selected for the first molar extraction treatment group (U6 group). Only those patient records that satisfied the inclusion and exclusion criteria were selected for the final study sample. Inclusion criteria were: Caucasian ethnic origin, late pubertal stage (CS3/CS4) [17], Angle Class II division 1 malocclusion, bilateral Class II molar relationship ≥½ premolar width, overjet ≥ 4 mm, no missing teeth or agenesis, permanent dentition, treatment with 1-stage full fixed appliance, extraction of maxillary first premolars or first permanent molars, no lower arch extraction and good general health with the absence of craniofacial syndromes or other craniofacial anomalies.

In the U4 group, maxillary first premolars were extracted before the onset of orthodontic therapy. Subsequently, a preadjusted fixed orthodontic straight-wire appliance (Roth prescription, 0.022 × 0.028-in slot) was bonded with an initial 0.014″ or 0.016″ NiTi wire, followed by a 0.017 × 0.025″ NiTi wire and a 0.019 × 0.025″ SS wire with sliding mechanics (elastic o-chains) and Class II elastics for space closure. The mean duration of active treatment was 2.1 ± 0.3 years.

In the U6 group, maxillary first molars were extracted before the onset of orthodontic therapy. Treatment consisted of three phases: Class II correction, space closure managing torque and finishing. Second upper molars were connected by a transpalatal arch. Low-friction light-wire brackets were bonded. Australian wire (0.16″) with anchor bends mesial to maxillary second molars and lower first molars were inserted. Class I and/or Class II elastics were used for Class II correction. Prior to space closure, maxillary premolars were included in the appliances and a 0.18″ Australian wire inserted. The mean duration of active treatment was 2.5 ± 0.6 years.

### 2.2. Cephalometric Analysis

**Figure 1:**
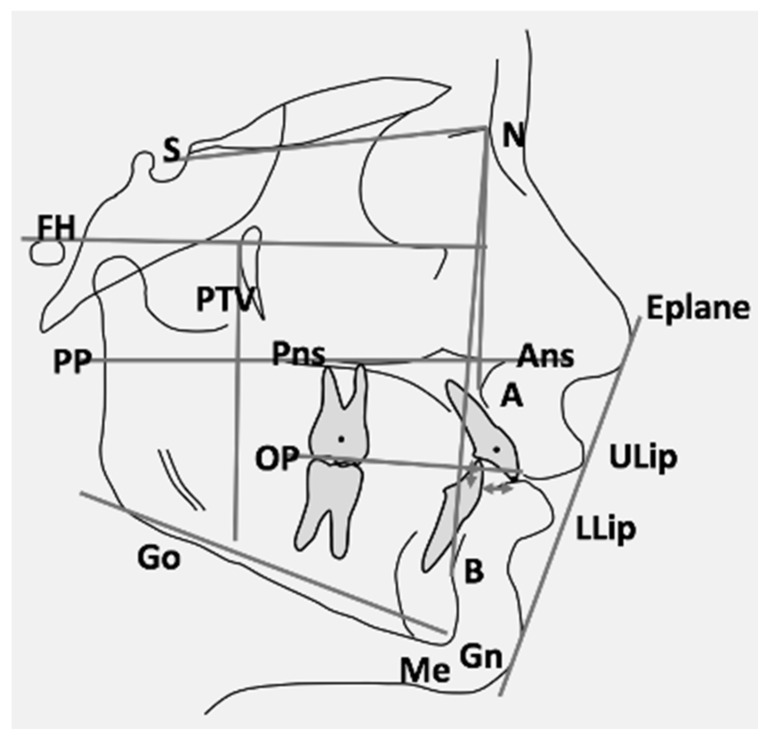
Graphical representation of reference points and planes used for the cephalometric analysis (S, Sella; N, Nasion; FH, Frankfurt plane; PP, palatal plane; PTV, pterygoid vertical; Pns, posterior nasal spine; Ans, anterior nasal spine; OP, occlusal plane; A, A point; B, B point; Go, Gonion; Me, Menton; Gn, Gnation; Eplane, esthetic plane; ULip, upper lip; LLip, lower lip).

Standardised lateral cephalograms were available at baseline (T0) and after active treatment (T1) for all patients. Enlargement factors were similar among radiographic units (about 8%); thus, no correction for enlargement was made analysing the radiographs. The same examiner (M.F.) traced all X-rays, and 9 linear and 11 angular measurements were calculated. To analyse the error of the method, cephalometric tracings were repeated for 10 subjects randomly selected from the two groups. Figure 1 represents all references’ points and planes used for the cephalometric analysis.

### 2.3. Method Error and Statistical Analysis

To quantify the method error for both linear and angular measurements, the method of moments (MME) variance estimator was used. Therefore, the mean error and 95% confidence intervals (CIs) between repeated recordings were calculated using the MME variance estimator.

Sample size was calculated, considering as the primary outcome overjet reduction on the data of a preliminary selected pilot sample of 6 patients (difference between means = 1.37; highest SD = 1.45). To retrieve β = 0.80 with α set at 0.05, a sample of at least 18 subjects per group was necessary. This means that the available sample size was sufficient.

SPSS software, version 22.0 (SPSS^®^ Inc., Chicago, IL, USA) was used for the statistical analysis. The Shapiro–Wilk test revealed a normal distribution of data; therefore, parametric tests were employed. Student’s *t*-test was used to compare the baseline data (T0) of the treated and control groups. Means and standard deviations (SDs) were calculated for both groups. A paired *t*-test was used to analyse changes between T0 and T1 within each group, and Student’s *t*-test to compare differences between groups at T1. The level of significance was set at *p* < 0.05.

## 3. Results

The intra-observer measurement error for linear measurements was on average 0.75 mm (range 0.6–0.9 mm), and 0.95° for angular measurements (range 0.7–1.2°).

At baseline T0 (Table 1), 6 out of 20 cephalometric variables showed a significant difference between the two groups. Most of the differences were found in the skeletal variables. It is important to notice that U6 SN^GoGn was within normal limits.

In the U4 group (Table 2), 11 out of 20 cephalometric variables showed a significant difference between T0 and T1. Most of the differences were found in the dental and soft tissue variables.

In the U6 group (Table 3), 16 out of 20 cephalometric variables displayed a significant difference between T0 and T1. In addition, in this case most of the differences were found in dental and soft tissue variables.

An intergroup comparison between the U4 and U6 group showed several statistically significant changes regarding dental-linear measurements, and a significant difference in the final position of the upper lip (Table 4).

### 3.1. Skeletal Changes

No statistically significant differences (*p* > 0.05) between groups were detected for the sagittal, and vertical skeletal and occlusal plane changes during treatment; although, SNA (U6 group: −2.34 ± 1.95°; U4 group: −1.47 ± 1.91°) and ANB (U6 group: −1.95 ± 1.65°; U4 group: −1.08 ± 1.98°) showed a clinically relevant decrease in both groups. SNB (U6 group: −0.39 ± 1.34°; U4 group: −0.36 ± 1.03°) (Table 4).

### 3.2. Dental Changes

The changes in dental angular variables during treatment did not show significant differences between groups, while three out of six dental linear variables differed between groups (Table 4). The overjet reduction was significantly larger for the U6 group than for the U4 group (U6 group: −4.86 ± 1.62 mm; U4 group: −3.27 ± 1.90 mm; *p* = 0.001), while the overbite reduction did not differ between groups (U6 group: −1.55 ± 2.41 mm; U4 group: −1.96 ± 1.59 mm). The distance from the PTV-maxillary second molar centroid increased more in the U6 group than in the U4 group (U6 group: 6.66 ± 5.00 mm; U4 group: 3.66 ± 2.20 mm; *p* = 0.012), indicating a larger mesialization of the upper second molars in the U6 group.

### 3.3. Soft Tissues and Profile Changes

The ULip-E Plane showed a statistically significant bigger reduction in U6 than in U4 (U6 group: −2.98 ± 1.65 mm; U4 group: −1.93 ± 1.57 mm; *p* = 0.021), while for the LLip-E Plane reduction, no significant differences were found between groups (U6 group: −1.95 ± 1.91 mm; U4 group: −1.61 ± 2.31 mm) (Table 4).

## 4. Discussion

The aim of this study was to compare skeletal, dental and soft tissues changes in Class II patients treated orthodontically with fixed appliances and either upper first premolar extraction or upper first molar extraction. The initial hypothesis was rejected, suggesting that there were some statistically significant differences among these treatment strategies. These differences were mainly found on the dental level. As could be expected, the sagittal skeletal effect was comparable for both groups as both treatments did not aim at modifying the growth pattern. The effect on the upper lip profile was larger in the molar extraction group.

The analysis of the baseline data showed that the U6 group had cephalometric characteristics that pointed to a more severe Class II division 1 malocclusion than in the U4 group. This should be taken into consideration when interpreting the results.

Earlier studies and systematic reviews analysed skeletal, dental and profile changes after premolar extraction, whereas only a few focused on first molar extraction [18]. A systematic review by Kouvelis et al. [19] found no significant differences between premolar extraction (Ex) and non-extraction treatment (Nonex) regarding vertical parameters in ten out of fourteen studies. Significant increases were found in Nonex for N-Me (Ex: +1.5 mm; Nonex: +5.5 mm; *p* < 0.05) [15] and for SN^GoGn (Ex: −0.9°; Nonex: +0.8°; *p* < 0.05) [16], but without concurrent changes in other vertical measurements. Two other studies showed opposite effects on N-Me (Ex: +2.3 mm; Nonex: +0.9 mm; *p* < 0.05) [20] and FMA (Ex: +0.3°; Nonex: −2.0°; *p* < 0.05) [21], indicating that vertical parameters increased more in extraction cases. The present study also showed a slight increase in ANS-Menton and in SN^GoGn in both groups; although, differences among groups were not statistically significant. These results suggest that a maxillary first molar extraction Class II treatment aiming to reduce the vertical dimension is not effective. The same results were found after cervical headgear treatment [22], and after Pendulum use followed by fixed appliance that caused a temporary increase in the vertical facial dimension during the active treatment phase, which returned to initial values during the post-retention period [23]. In contrast, other studies, including a literature review about non-compliance intraoral distalising appliances anchored on the dentition, showed an increase in the vertical facial dimension [24,25].

Regarding the skeletal sagittal correction, a systematic review by Janson showed a significant reduction of the ANB angle of 1.56° (*p* < 0.001) in treated Class II Nonex patients compared with untreated Class II subjects. Class II treated with extraction of two maxillary premolars or four premolars produced an estimated mean reduction in ANB [26]. However, the quality of the evidence was low, as only one study about Class II treatment with upper premolar extractions had an untreated Class II control group [27]. In accordance with these findings, we found that the ANB angle in both groups was significantly smaller at the end of treatment. Moreover, the reduction was bigger in the U6 group compared to the U4 group, but this difference was not statistically significant. According to the pubertal stage and Class II late treatment, no statistically nor clinically significant SNB modifications were detected.

Class II extraction treatment carries the risk of proclination of the lower incisors and loosing upper incisors’ root torque control with consequent poor overjet and overbite correction, compromised lateral intercuspation and incomplete closure of the extraction spaces [28]. Booij et al. showed, in Class II patients treated with maxillary first molar extractions, that the overjet reduction of 5.2 mm in total was achieved by skeletal changes (1.7 mm) expressed as dorsal movement of point A and forward movement of point Pg and by dental changes (3.5 mm) as retrusion of upper incisors and protrusion of lower incisors [15]. The present results agree with the previously discussed ones, as the inclination of upper incisors over SN reduced indeed.

In the present study, we found a larger overjet reduction in the U6 group compared to the U4 group. The explanation could be that upper molar extractions provided more space in the dental arch which ensured greater overjet correction than premolar extractions [13]. It is not possible to exactly assess which dental changes contributed to solve the overjet, because no dental values about mandibular incisors were considered. Booij et al. found that space closure in the upper jaw was achieved especially by the mesialization of the second molars [16]. This is consistent with the current study, which revealed an increase in the distance of the PTV-maxillary second molar centroid in both groups. The greater increase in the U6 group than in the U4 group could be explained by the fact that the extraction spaces in the U6 group were mainly used for managing a medium-minimum anchorage for Class II correction, whereas the extraction spaces in the U4 group were mainly used for resolving crowding. Booij et al. compared the skeletal effects of upper first molar extraction with non-extraction treatment with a Herbst-multibracket appliance showing a larger decrease of SNA in the extraction group, a bigger increase of SNB in the Herbst group and a slight decrease in the extraction group [16]. The current study found comparable results.

It is widely recognised that changes in skeletal structures are reflected in overlying soft tissues. Actually, soft tissue protrusion and upper lip position express the amount of maxillary and dental protrusion. However, the correlation between cephalometric parameters and corresponding soft tissues landmarks is weak [29]. Affecting the soft tissue profile is possible with a fixed appliance treatment. A systematic review showed a statistically significant increase in the nasolabial angle and retrusion of upper and lower lips in a 2- or 4-premolars extraction protocol [30]. These results suggested a decrease in the ULip-E Plane which was also found in the current investigation, with a greater reduction in the U6 group than in the U4 group. These results showed that upper first molar extraction affected the soft tissue profile appearance more than upper premolar extraction. Luckily, the baseline data showed that the upper lip protrusion was bigger in the U6 group compared to U4 group at the start of treatment. Similar upper and lower lip retraction, increase of the nasolabial angle and profile convexity were demonstrated by Konstantonis et al. [31], even though the quality of evidence was rather low in their systematic review.

Extraction treatment for Class II malocclusion does not lead to a worsening of the face profile if the therapeutic indication is correct, and it is essential to consider the accordance between diagnosis and treatment. Lip structure has an influence on lip response to incisor retraction regarding the E Plane. A correlation between soft tissue and osseous changes after incisor retraction have been previously explained by soft tissue thickness [32]. For this reason, extraction of maxillary premolars or molars should be recommended only in patients with an increased OVJ, adequate lip thickness and distance from the E Plane, and a protruded profile that becomes straighter after treatment.

The present study was a retrospective study in two orthodontic offices. Ideally, this research question should be investigated in a randomized controlled trial that prevents selection bias and enables the standardization of treatment methods. The U6 group presented more severe Class II and patients were younger than the U4 group. This should be taken into account when evaluating the results. The treatment outcome was analyzed from the orthodontist’s perspective. Patient reported outcome measures (PROMs) were not available for this sample; therefore, the present study does not provide information on the patients’ perspectives on orthodontic treatment with extraction of maxillary first premolars or molars. Finally, data recollected relate exclusively to a Caucasian population, and this is certainly a further limitation of the present study; further research may be carried out on other ethnic origins to compare our results with.

## 5. Conclusions

Based on the results of the present study, the following conclusions can be drawn:

- Orthodontic treatment with maxillary first premolar or first molar extraction produces comparable vertical skeletal results. Therefore, an increased SN^GoGn angle should not be considered the discriminating factor for choosing a first molar extraction treatment over a first premolar extraction treatment.
- The significantly larger reduction of upper lip protrusion and overjet after maxillary first molar extraction compared to first premolar extraction suggests preferring molar extraction treatment in the case of a Class II malocclusion with a protrusive soft tissue profile and increased overjet.

## Figures and Tables

**Table 1:** Baseline data (T0) for the cephalometric variables; * *p* < 0.05.

Measurements T0	U6		U4		*p*
	Mean	SD	Mean	SD	
Soft tissue					
ULip-E Plane (mm)	−0.83	2.23	−1.59	2.59	0.239
LLip-E Plane (mm)	−0.11	2.33	−0.99	2.78	0.2
Skeletal					
SNA (°)	83.04	3.16	81.53	3.83	0.108
SNB (°)	75.58	2.83	76.97	2.88	0.078
ANB (°)	7.47	1.84	4.53	2.40	<0.001 *
SN^PP (°)	5.49	2.73	5.95	2.90	0.549
SN^GoGn (°)	33.27	1.84	29.98	3.52	<0.001 *
PTV-A point (mm)	50.37	2.80	48.13	3.44	0.009 *
PTV-B point (mm)	39.74	4.55	41.49	3.87	0.143
ANS Menton (mm)	66.54	3.41	63.74	4.28	0.008 *
Occlusal plane					
SN^OP (°)	19.72	3.53	18.27	3.91	0.152
OP^GoGn (°)	13.56	3.23	11.69	2.70	0.029 *
Dental-angular					
SN^maxillary incisor (°)	105.72	6.25	105.51	8.36	0.913
SN^maxillary second molar (°)	60.92	5.73	61.40	6.77	0.776
Dental-linear					
PTV-maxillary incisor centroid (mm)	52.69	3.76	51.44	3.84	0.229
PTV-maxillary second molar centroid (mm)	10.44	3.01	10.40	2.75	0.953
PP-maxillary incisor tip (mm)	28.04	1.74	27.43	3.00	0.331
PP-maxillary second molar centroid (mm)	12.69	2.65	12.00	6.61	0.575
Overjet (mm)	7.75	1.63	5.82	1.73	<0.001 *
Overbite (mm)	1.97	2.61	2.20	1.76	0.727

**Table 2:** Cephalometric variables at T0 and T1 (means and SD) for the U4 group and results of the paired sample *t*-test (*p*-values); * *p* < 0.05.

Cephalometric Variables U4	T0		T1		*p*
	Mean	SD	Mean	SD	
Soft tissue					
ULip-E Plane (mm)	−1.59	2.59	−3.52	1.96	<0.001 *
LLip-E Plane (mm)	−0.99	2.78	−2.60	1.61	<0.001 *
Skeletal					
SNA (°)	81.53	3.83	80.06	3.67	<0.001 *
SNB (°)	76.97	2.88	76.61	2.76	0.13
ANB (°)	4.53	2.40	3.45	2.52	0.02 *
SN^PP (°)	5.95	2.90	6.18	3.05	0.40
SN^GoGn (°)	29.98	3.52	30.30	2.91	0.53
PTV-A point (mm)	48.13	3.44	47.96	3.94	0.65
PTV-B point (mm)	41.49	3.87	41.77	4.60	0.60
ANS Menton (mm)	63.74	4.28	67.00	4.94	<0.001 *
Occlusal plane					
SN^OP (°)	18.27	3.91	18.89	3.91	0.25
OP^GoGn (°)	11.69	2.70	11.42	2.73	0.54
Dental-angular					
SN^maxillary incisor (°)	105.51	8.36	102.35	5.68	0.12
SN^maxillary second molar (°)	61.40	6.77	70.12	4.64	<0.001 *
Dental-linear					
PTV-maxillary incisor centroid (mm)	51.44	3.84	49.04	3.91	<0.001 *
PTV-maxillary second molar centroid (mm)	10.40	2.75	14.05	3.48	<0.001 *
PP-maxillary incisor tip (mm)	27.43	3.00	27.64	2.75	0.64
PP-maxillary second molar centroid (mm)	12.00	6.61	16.00	2.82	0.01 *
Overjet (mm)	5.82	1.73	2.55	0.93	<0.001 *
Overbite (mm)	2.20	1.76	0.24	0.84	<0.001 *

**Table 3:** Cephalometric variables at T0 and T1 (means and SD) for the U6 group and results of the paired sample *t*-test (*p*-values); * *p* < 0.05.

Cephalometric Variables U6	T0		T1		*p*
	Mean	SD	Mean	SD	
Soft tissue					
ULip-E Plane (mm)	−0.83	2.23	−3.81	2.21	<0.001 *
LLip-E Plane (mm)	−0.11	2.33	−2.06	2.32	<0.001 *
Skeletal					
SNA (°)	83.04	3.16	80.70	3.50	<0.001 *
SNB (°)	75.58	2.83	75.18	2.90	0.08
ANB (°)	7.47	1.84	5.52	2.01	<0.001 *
SN^PP (°)	5.49	2.73	5.58	3.14	0.77
SN^GoGn (°)	33.27	1.84	34.03	2.66	0.02 *
PTV-A point (mm)	50.37	2.80	49.59	2.93	0.04 *
PTV-B point (mm)	39.74	4.55	39.71	4.48	0.94
ANS Menton (mm)	66.54	3.41	70.74	4.19	<0.001 *
Occlusal plane					
SN^OP (°)	19.72	3.53	21.24	3.82	<0.001 *
OP^GoGn (°)	13.56	3.23	12.79	3.69	0.12
Dental-angular					
SN^maxillary incisor (°)	105.72	6.25	102.95	4.50	0.01 *
SN^maxillary second molar (°)	60.92	5.73	70.49	5.51	<0.001 *
Dental-linear					
PTV-maxillary incisor centroid (mm)	52.69	3.76	50.00	3.24	<0.001 *
PTV-maxillary second molar centroid (mm)	10.44	3.01	17.10	5.74	<0.001 *
PP-maxillary incisor tip (mm)	28.04	1.74	29.13	1.95	<0.001 *
PP-maxillary second molar centroid (mm)	12.69	2.65	15.89	2.37	<0.001 *
Overjet (mm)	7.75	1.63	2.89	0.78	<0.001 *
Overbite (mm)	1.97	2.61	0.42	0.87	<0.001 *

**Table 4:** Comparison of the increments between U6 and U4 group after treatment; * *p* < 0.05.

Measurements T1	U6		U4		*p*
	Mean	SD	Mean	SD	
Soft tissue					
ULip-E Plane (mm)	−2.98	1.65	−1.93	1.57	0.021 *
LLip-E Plane (mm)	−1.95	1.91	−1.61	2.31	0.551
Skeletal					
SNA (°)	−2.34	1.95	−1.47	1.91	0.102
SNB (°)	−0.39	1.34	−0.36	1.03	0.912
ANB (°)	−1.95	1.65	−1.08	1.98	0.076
SN^PP (°)	0.09	1.79	0.22	1.20	0.756
SN^GoGn (°)	0.76	1.99	0.33	2.33	0.458
PTV-A point (mm)	−0.77	2.30	−0.17	1.66	0.291
PTV-B point (mm)	−0.04	3.25	0.28	2.38	0.698
ANS Menton (mm)	4.20	2.30	3.26	2.75	0.165
Occlusal plane					
SN^OP (°)	1.52	2.82	0.61	2.38	0.216
OP^GoGn (°)	−0.76	2.98	−0.27	1.96	0.496
Dental-angular					
SN^maxillary incisor (°)	−2.77	6.36	−3.16	8.89	0.849
SN^maxillary second molar (°)	9.57	6.95	8.72	6.69	0.653
Dental-linear					
PTV-maxillary incisor centroid (mm)	−2.70	2.58	−2.40	2.04	0.651
PTV-maxillary second molar centroid (mm)	6.66	5.00	3.66	2.20	0.012 *
PP-maxillary incisor tip (mm)	1.09	1.52	0.20	2.00	0.061 *
PP-maxillary second molar centroid (mm)	3.20	2.86	4.00	6.07	0.493
Overjet (mm)	−4.86	1.62	−3.27	1.90	0.001 *
Overbite (mm)	−1.55	2.41	−1.96	1.59	0.49

## Data Availability

The data presented in this study are available on request from the corresponding author. The data are not publicly available due to privacy limitations.
